# Modulation of Hippocampal GABAergic Neurotransmission and Gephyrin Levels by Dihydromyricetin Improves Anxiety

**DOI:** 10.3389/fphar.2020.01008

**Published:** 2020-07-09

**Authors:** Joshua Silva, Amy S. Shao, Yi Shen, Daryl L. Davies, Richard W. Olsen, Daniel P. Holschneider, Xuesi M. Shao, Jing Liang

**Affiliations:** ^1^ Titus Family Department of Clinical Pharmacy, University of Southern California School of Pharmacy, Los Angeles, CA, United States; ^2^ Molecular & Medical Pharmacology, David Geffen School of Medicine at UCLA, Los Angeles, CA, United States; ^3^ Department of Neurobiology, NHC and CAMS Key Laboratory of Medical Neurobiology, Zhejiang University School of Medicine, Hangzhou, China; ^4^ Psychiatry and The Behavioral Sciences, University of Southern California, Los Angeles, CA, United States; ^5^ Neurobiology, David Geffen School of Medicine at UCLA, Los Angeles, CA, United States

**Keywords:** anxiety, dihydromyricetin (DHM), GABA_A_R, gephyrin, social isolation

## Abstract

Anxiety disorders are the most common mental illness in the U.S. and are estimated to consume one-third of the country’s mental health spending. Although anxiolytic therapies are available, many patients exhibit treatment-resistance, relapse, or substantial side effects. An urgent need exists to explore the underlying mechanisms of chronic anxiety and to develop alternative therapies. Presently, we identified dihydromyricetin (DHM), a flavonoid that has anxiolytic properties in a mouse model of isolation-induced anxiety. Socially isolated mice demonstrated increased anxiety levels and reduced exploratory behavior measured by elevated plus-maze and open-field tests. Socially isolated mice showed impaired GABAergic neurotransmission, including reduction in GABA_A_ receptor-mediated extrasynaptic tonic currents, as well as amplitude and frequency of miniature inhibitory postsynaptic currents measured by whole-cell patch-clamp recordings from hippocampal slices. Furthermore, intracellular ATP levels and gephyrin expression decreased in anxious animals. DHM treatment restored ATP and gephyrin expression, GABAergic transmission and synaptic function, as well as decreased anxiety-like behavior. Our findings indicate broader roles for DHM in anxiolysis, GABAergic neurotransmission, and synaptic function. Collectively, our data suggest that reduction in intracellular ATP and gephyrin contribute to the development of anxiety, and represent novel treatment targets. DHM is a potential candidate for pharmacotherapy for anxiety disorders.

## Introduction

Anxiety disorders are a group of mental disorders and a leading cause of disability in Western societies (Craske et al., 2017). Anxiety disorders, including generalized anxiety disorder, panic disorder, social anxiety disorder, obsessive-compulsive disorder, post-traumatic stress disorders, and phobias, typically have an early onset, run a chronic or relapsing course, cause substantial personal distress, impair social and occupational function, reduce quality of life, and impose a substantial economic burden (Kessler et al., 2005; Toghanian et al., 2014). These disorders cost the U.S. more than $42 billion a year, comprising almost one-third of the country’s total mental health bill allotment (Sinoff and Werner, 2003; Merikangas et al., 2010). Furthermore, anxiety disorders, with generalized anxiety disorder being the most common, are found in both younger and older adults, suggesting that these disorders can manifest throughout the lifespan (Lenze and Wetherell, 2011). Anxiety disorders often are comorbid with major depression, bipolar disorder, schizophrenia, substance misuse, and physical illness, and are associated with increased risks of suicidal behavior and downstream cognitive decline (DeVane et al., 2005; Banks et al., 2014).

Approximately 20–32% of patients suffering from anxiety disorders are adequately treated with available therapies that have 50–60% response rates in clinical trials (Roy-Byrne, 2015). Unfortunately, many of these therapeutics can be ineffective in the clinic due to treatment resistance, patient adherence, and other exogenous factors (DeVane et al., 2005; Konnopka et al., 2009; Roy-Byrne, 2015), thereby resulting in failed responses in many patients (Batelaan et al., 2017). Benzodiazepines (BZs) are established first-line treatments, but many side effects such as drowsiness, falls, confusion, memory impairment, incoordination (especially in the elderly and children), dependence, and their potential for substance abuse and addiction significantly limit their use (Cloos and Ferreira, 2009; Davidson, 2009). Due to these side effects and risks, alternatives such as selective serotonin reuptake inhibitors and serotonin/norepinephrine reuptake inhibitors have emerged as additional therapies for the treatment of anxiety disorders. However, similar to benzodiazepine (BZ) therapies, many patients remain treatment-resistant, and side effects, including agitation, gastrointestinal symptoms, and increased suicidal thoughts, often limit patient compliance (Nutt, 1997; Sánchez and Meier, 1997; Rickels and Rynn, 2002). Other agents for the treatment of anxiety disorders include atypical neuroleptics, but dosing is limited by sedation, fatigue, and possible extrapyramidal symptoms (Hyun et al., 2010). In addition, the chronic treatment with the above-mentioned anxiolytics can result in a loss of efficacy and require increased dosing for maintained therapeutic effects. Clearly, new treatment strategies are needed that are capable of effectively and safely treating anxiety disorders with consistent effects, reduced rates of relapse, and fewer adverse side effects.

We have demonstrated that dihydromyricetin [(2R,3R)-3,5,7-trihydroxy-2-(3,4,5-trihydroxyphenyl)-2,3-dihydrochromen-4-one (DHM), a flavonoid component of *Ampelopsis grossedentata* (Shen et al., 2012; Liang et al., 2014a) is highly effective in counteracting acute alcohol (ethanol/EtOH) intoxication, reducing alcohol consumption, as well as counteracting alcohol withdrawal symptoms, including withdrawal-related anxiety (Shen et al., 2012; Liang et al., 2014b). Additionally, prior work by our group has demonstrated the anxiolytic effects of DHM in Alzheimer’s disease (AD) mouse models (TG2576 and TG-SwDI) as measured in behavioral studies using the elevated plus-maze and open field (Liang et al., 2014a).

Clinical evidence indicates that altered GABAergic neurotransmission contributes to the pathophysiology of anxiety disorders in humans (Rudolph and Knoflach, 2011). Therefore, modifying GABA_A_ receptor (GABA_A_Rs) activity is one underlying mechanism for regulating anxiety (Shekhar et al., 1990; Shekhar, 1993; Rudolph and Knoflach, 2011). We have demonstrated that DHM can modulate GABAergic transmission (Shen et al., 2012; Liang et al., 2014a) and therefore has the potential to regulate anxiety-like behavior via its action on GABAergic receptors. At the cellular level, we have found that DHM inhibits the acute and chronic effects of alcohol on GABA_A_Rs (Shen et al., 2012; Liang et al., 2014b). Therefore, the activity of DHM on GABA_A_Rs provides one possible mechanism for its activity and role in anxiolysis (Liang et al., 2014b).

To further understand alternate pharmacological mechanisms of DHM as an anxiolytic, we utilized a social isolation model of mice that induces anxiety via reduced social interaction as a chronic stressor. This model of social isolation results in long-lasting effects on behavior and brain structure in rodents (Koike et al., 2009; Berry et al., 2012). However, this study was interested in understanding the pharmacological responses of DHM in adult mice with the chronic stress of social isolation that has also been linked to anxiety-like responses (Ieraci et al., 2016). The primary goal of this study was to investigate the utility of DHM as an anxiolytic in comparison to other GABA_A_R modulating anxiolytics for chronic anxiety disorders, as well as to continue the inquiry into its underlying neurobiological and cellular mechanisms.

## Methods

### Overview

Eight-week old male C57BL/6 mice (Charles River Laboratories, Hollister, CA) were housed in the vivarium under a 12 h light/dark cycle with direct bedding and free access to food and water. All animal experiments were performed according to the protocols approved by the University of California (UCLA) Institutional Animal Care and Use Committee, and all methods were carried out in accordance with relevant guidelines and regulations. Animals were habituated to the vivarium for 2 d before beginning experimentation. Tissue biochemical analyses were conducted at the University of Southern California (USC).

### Social Isolation

Social isolation is known to elicit anxious and depressive behaviors in rodents (Pinna et al., 2004; Pinna et al., 2006; Cryan and Sweeney, 2011; Hershenberg et al., 2014). These protocols were modified to induce stress associated with social isolation by using opaque walled cages and depriving the animals of toys/objects. Furthermore, we investigated the anxiety-like behaviors both 4- and 6-weeks post-social isolation to determine behavioral responses comparable to the established 4–6 week isolation that results in anxiety (Pinna et al., 2006). We used these time points to determine potential therapeutic effects of DHM. Group-housed mice were housed with the standard 3–4 mice per cage. Isolated mice were singly housed with opaque walls without human handling except to change cages once per week.

1) Group of group-housed mice without any drug administration for 2 weeks, and then were given daily administration of sucrose agar as vehicle for an additional 2 weeks (G2+Veh2).

2) Group of group-housed mice without any drug administration for 2 weeks, and then were given daily administration of DHM in sucrose+agar (vehicle) for an additional 2 weeks (G2+D2, 2 mg/kg DHM).

3) Isolated mice without any drug administration for 2 weeks, and then were given daily administration of vehicle for an additional 2 weeks for a total isolation period of 4 weeks (Iso2+Veh2).

4) The isolated mice without any drug administration for 2 weeks, and then were given daily administration of DHM in vehicle for an additional 2 weeks for a total isolation period of 4 weeks (Iso2+D2).

5) The isolated mice without any drug administration for 4 weeks, and then were given daily administration of DHM in vehicle for an additional 2 weeks for a total isolation period of 6 weeks (Iso4+Veh2).

6) The isolated mice without any drug administration for 4 weeks, and then were given daily administration of diazepam in vehicle for an additional 2 weeks for a total isolation period of 6 weeks (Iso4+DZ2; 10 mg/kg).

Isolated mice are kept singly housed during the 2 weeks of drug treatment. DHM was administered starting at both time points (2- and 4-week) to determine anxiolytic properties, and anxiety-like behavior, meanwhile comparing effects against DZ starting at week 4 to determine responses in comparison to the reference drug. Therefore, all mice were either group housed (control) for 2 weeks or isolated for either 2- or 4-weeks before an additional 2-weeks of drug treatment to assess time-dependent responses with isolation against group housing.

### Drug Preparations

DHM (HPLC purified ≥ 98%, Master Herbs Inc., Pomona, CA) and DZ (10 mg/kg; Sigma-Aldrich, St. Louis, MO) was administered orally (Liang et al., 2014a), once per day (2 mg/kg) for 2-weeks. To prepare the DHM agar, 3% agar was prepared with water, heated to ~90 °C to dissolve the agar, then DHM + 5% sucrose or DZ + 5% sucrose or 5% sucrose only were added and mixed until cooled and solidified. Agar was prepared for the mice by cutting it into cubes of 0.5 x 0.5 x 0.5 cm each. 10 mg/kg dose of DZ has been shown to be an effective anxiolytic in rats in the conditioned fear paradigm (Bachmanov et al., 2002; Hershenberg et al., 2014) with minimal to no effect on locomotor activity (Chen et al., 2004; Olsen and Sieghart, 2008; Kou et al., 2012). Furthermore, DZ was included as the drug of comparison against DHM to evaluate responses in a modified social isolation protocol (Wongwitdecha and Marsden, 1996; Varty et al., 2006; Lander et al., 2017) that has previously been reported to produce variable effects of DZ in social isolation models.

Drug Administration

Every evening (8 PM), all food from the cages of each mouse was removed, and an agar cube was placed in the cage for each mouse. The mouse was observed to ensure it ate the agar cube, which ranged from 30 to 90 min. Afterward, 4 g of regular rodent food (the recommended daily amount for an adult mouse; [Bachmanov et al., 2002]) was placed in the cage for each mouse for the rest of the day. To ensure each mouse of the group housing mice received one cube, they were isolated, fed, and then returned to group housing. D2 mice received sucrose-DHM agar cubes daily for 2-weeks, and DZ2 mice received sucrose-DZ agar cubes daily for the 2-weeks.

### Behavioral Testing

Anxiety-like behavior was tested 24 h after the last drug/vehicle treatment, a time point shown to have continued DZ-mediated anxiolytic effects in alcohol withdrawn C57Bl/6J mice (Dominguez et al., 2018), at the end of the 4-week or 6-week time points in the following evening (8 PM.; in the dark phase of 12/12-h light/dark cycle) using anxiety tests reliant on ethologically appropriate behavior and sensitive to “state” anxiety measurements. Mice are nocturnal animals. Behavior testing was conducted during their active time to ensure accurate behavioral responses and minimize interference of their circadian cycle. Behavioral tests were performed under indirect red lighting by video camera. Indirect red lighting was used to better assess parameters of anxiety without influencing mouse behavior (i.e., reduced activity in the open field test) and stress, which has been observed with direct light or bright light stimuli (McReynolds et al., 1967; Kapogiannatou et al., 2016; Peirson et al., 2018). Investigators were blind to mice groups when conducting behavioral analyses.

#### *Elevated Plus Maze*

The elevated plus maze was conducted following a previously published protocol (Kou et al., 2012; Liang et al., 2014b). The elevated plus-maze apparatus was made of opaque plastic 0.6 cm thick. It comprised two open arms 25 x 8 cm (LxW) across from each other and perpendicular to two closed-arm 25x8 cm with a center platform of 8x8 cm. The closed arms had a 20 cm high wall that enclosed the arms. The wall and the floor of the closed arms were black, and the floor of the open arms was white. The elevated plus maze was elevated 50 cm above the floor of the laboratory. Throughout the test, each animal was placed in the center of the maze facing an open arm and allowed to explore for a 5 min session. The behavior was recorded by a ceiling-mounted camera. Entry into an arm of the maze was defined by the placement of at least three paws into that compartment. The following measures were scored: number of entries into open arms, closed arms, or center platform and time spent in each of these areas. All scoring was performed off-line in a double-blind manner.

#### Open Field Test

The open field test was conducted following a previously published protocol (Chen et al., 2004; Liang et al., 2014a). The open field chamber measured 50 cm (length) x 50 cm (width) x 38 cm (height) and was made from a white acrylic plastic sheet. Four by 4 grid lines were drawn to divide the floor into 10 x 10 cm squares, and an additional 20 x 20 cm square zone was drawn in the center. Mouse activity was assessed as previously reported (Chen et al., 2004) in open field for 10 min. The following parameters: initial time (the time from when the mouse was first placed into the center of the apparatus to the time the mouse start moving), tail up time (the time of the mouse lift the tail up), the time spent in the central zone, spent in the four corner square grid, path length (cm) traveled in the apparatus (determined by measuring the distance of the nose of the mouse relative to the 10X10 cm square grid lines on the floor of the open field chamber), and the numbers of times of rears were summed for each animal during the 10-min test. All scoring was conducted manually in a double-blind manner, with each recording being observed three times to minimize error.

### Western Blots for Gephyrin

Gephyrin protein expression in mice was determined via Western blot analysis. Hippocampus from the right hemisphere was homogenized in pre-cooled Tris-EDTA extractant (0.1 M Tris-acetate buffer + 2 mM EDTA, pH 7.75) using a Branson Digital Sonifier 150 ultrasonic tissue disruptor-homogenizer (Emerson., St. Louis, MO). The homogenate was centrifuged at 10,000 x g for 10 min in a refrigerated centrifuge at 4°C and supernatant was collected. Protein supernatant was quantified using the BCA Protein Assay kit (Pierce Biotechnology, Rockford, IL) according to the manufacturer’s instructions. Fifty µg of proteins were separated on a 10 % sodium dodecyl sulfate-polyacrylamide gel electrophoresis and transferred to PVDF membranes for Western blot analysis (Bio-Rad Laboratories, Hercules, CA). Transferred membrane was blocked with blocking buffer containing 5% skim milk (Bio-Rad, Hercules, CA) in 1X Tris-buffered saline with Tween 20 (Thermofisher) for 1 h and then incubated with anti-gephyrin monoclonal antibody (Cell Signaling, Beverly, MA) at 1:1,000 in 1X TBST overnight at 4°C. The membrane was washed three times with 1X TBST for 10 min and incubated with secondary antibody in 1X TBST for 1 h, and the images were visualized with enhanced chemiluminescence detection reagent and Chemi-Doc (Bio-Rad) imaging device.

### Hippocampal ATP Bioluminescence

Hippocampus from the left hemisphere was homogenized in pre-cooled Tris-EDTA extractant (0.1 M Tris-acetate buffer + 2 mM EDTA, pH 7.75) using a Branson Digital Sonifier 150 ultrasonic tissue disruptor-homogenizer (Emerson., St. Louis, MO). The homogenate was centrifuged at 10,000 x g for 10 min in a refrigerated centrifuge at 4°C and supernatant was collected. Aliquots of supernatant were re-adjusted to pH 7.8 (according to required pH for assay guidelines) with 320 µl of 2.5 M KOH and precipitate removed by a second centrifugation (10,000 g for 10 min). Aliquots of supernatant were transferred to a fresh tube, on ice, and 240 µl of Tris-HCL/EDTA (pH 7.75) was added. For supernatant ATP levels to be accurately assayed using the Sigma ATP Bioluminescent Kit, final supernatant pH levels were re-adjusted to pH 7.8 (Sigma-Aldrich, St. Louis, MO). ATP levels of isolated and grouped mouse brain extracts (n= 4 for each group) were measured by using 100 μl of supernatant with an ATP luciferin bioluminescence assay kit according to the manufacturer’s guidelines. ATP assay mix was diluted with 5 ml of sterile water and remained on ice and protected from light for 1 h to assure complete dissolution. One hundred µl of ATP assay mix was added to each well and incubated at room temperature for 3 min to allow for hydrolysis of endogenous ATP. Immediately after adding 100 μl of tissue homogenates, standards, or water controls with ATP assay mix, the sample was measured for luciferase light production. Luminescence was measured using the BioTek Synergy H1 Hybrid Multi-Mode Reader plate reader (BioTek, Winooski, VT). Relative luminescent units from four measurements were averaged.

### Electrophysiological Recordings

Electrophysiological recordings were conducted as previously described (Liang et al., 2004; Shen et al., 2012). Following the social isolation (or group housing) 2 or 4 weeks and drug administration for 2 weeks, mice were carried over into the electrophysiological recordings. At the time of the electrophysiological recordings, brain harvesting, and analysis, all mice were either 12- or 14- week-old. Mice were anesthetized with isoflurane, and the brain was then removed after decapitation. The brain areas containing the hippocampus were dissected rapidly and transferred to a chamber filled with ice-cold artificial cerebrospinal fluid (ACSF; in mM: 124 NaCl, 2 KCl, 1.25 KH_2_PO_4_, 2 MgSO_4_, 2 CaCl_2_, 26 NaHCO_3_, and 10 D-Glucose, pH 7.4, 300 mOsm). Transverse hippocampal slices (400 μm) were cut with a tissue slicer (VT 1200S, Leica) and incubated in oxygenated (95% O_2_/5% CO_2_) ACSF. Slices were allowed to recover for ~30 min in ACSF at room temperature. Hippocampal slices were then transferred to a recording chamber and perfused continuously with ACSF at 35°C bubbled with 95% O_2_/5% CO_2_ to ensure adequate oxygenation of slices. Whole-cell recordings were performed on dentate gyrus (DG) neurons. Neurons were identified under infrared differential interference contrast (IR-DIC) optics based on their location and morphology. Borosilicate glass (A-M system) pipettes (3–6 MΩ) were pulled with a horizontal pipette puller (P97, Sutter instruments) and were filled with the solution (in mM: 137 CsCl, 2 MgCl_2_, 1 CaCl_2_, 11 EGTA, 10 HEPES, and 3 ATP-Mg (pH 7.30). Pipettes were connected to the head stage of a Heka EPC 10 amplifier (Heka Elektronik), and fast and slow capacitance and series resistance compensations were carefully adjusted. GABA_A_R-mediated miniature inhibitory postsynaptic currents (mIPSCs) were pharmacologically isolated by adding 0.5 μM TTX, 40 μM APV (Abcam), 10 μM CNQX (Abcam), and 1 μM CGP54626 (Tocris) to the bath solution. The GABA_A_R-mediated extrasynaptic tonic currents (I_tonic_) was calculated as the difference in holding current between the pre-drug and application of picrotoxin (Liang et al., 2004a). Series resistance was normally less than 20 MΩ, and recordings exceeding 20% change in series resistance were terminated and discarded. Electrophysiological recordings were filtered at 2.0 kHz and digitized at 50 kHz.

### Detection and Analysis of mIPSCs and GABA_A_R-Mediated I_tonic_

The mIPSCs were detected (MiniAnalysis Program, Version 6.0.7, Synaptosoft Inc. Fort Lee, NJ) with threshold criteria of 8 pA amplitude and 20 fC areas. The frequency of mIPSCs was determined from all automatically detected events in a given 100 s recording period. For kinetic analysis, only single-event mIPSCs with a stable baseline, sharp rising phase (10–90 % rise time), and exponential decay were used. The kinetics of mIPSCs were obtained from analysis of the averaged chosen single events aligned with half rise time in each cell. Decay time constants were obtained by fitting a double exponential to the falling phase of the averaged mIPSCs. The I_tonic_ magnitudes were obtained as the difference of the averaged baseline current of a given recording period minus the averaged current in the presence of picrotoxin (50 µM) (Olsen and Sieghart, 2008; Shen et al., 2012).

### Data Analysis

Data are expressed as the mean ± SEM. One-way ANOVA, followed by multiple comparison analyses based on Holm-Sidak method, was used to determine significance levels for multiple groups. Specific details in statistical analyses are described for each figure in the figure legends. SigmaStat (Systat Software Inc., San Jose, CA) and Graph-Pad Prism 6.0 were used. Differences among groups were stated to be statistically significant when *p* ≤ 0.05.

## Results

### DHM Ameliorates Social Isolation-Induced Anxiety Behavior

**Figure 1:**
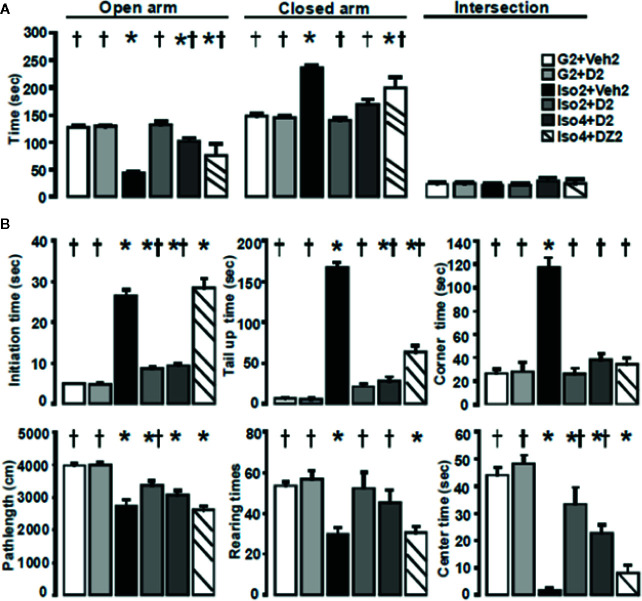
DHM ameliorates social isolation-induced anxiety. **(A)** Effects of social isolation and treatment with DHM on anxiety-like behavior as measured by the time (s) spent in the open, closed arms, and the area between cross arms (called “intersection”) of the elevated plus maze. one-way ANOVA followed by multiple comparison, Holm-Sidak method. For open arm, F(5, 30) = 26.42, p < 0.001. For close arms; F(5, 30) = 34.81, p < 0.001. For intersection, F(5, 30) = 0.50 p = 0.78. **(B)** Effects of social isolation and treatment with DHM on locomotor activity, exploratory behavior as measured by initial time (the time duration from when the mouse first placed into the center of the apparatus to start moving), tail up (the time of tail lifting), rearing (total number of times of rearings), path length (total distance of moving), center time (the total time duration the mouse stayed in the center 20 x 20 cm square), and corner (the total duration the mouse stayed in the 4 corner 10x10 cm squares) in the open field assay. One-way ANOVA followed by multiple comparison, Holm-Sidak method. For initial time, F(5, 30) = 108.35, p < 0.001. For tail up, F(5,30) = 184.4, P <0.001. For stay in corners F(5, 30) = 47.1, P < 0.001. For path length, F(5, 30) = 15.9, P < 0.001; For numbers of rearings, F(5, 30) = 7.74, P < 0.001. For stay in the center, P(5, 30) = 34.1, P < 0.001. *, *p* ≤ 0.05 vs. vehicle group housing control (G2+Veh2). †, *p* ≤ 0.05 vs. Iso2+Veh2, (n = 6/group).

Using a mouse model of social isolation stress (Dityatev and Bolshakov, 2005; Ieraci et al., 2016), we examined the anxiolytic effects of DHM with the elevated plus-maze and open field tests. Group-housed control mice (G2+Veh2) spent 128± 3 s of a total 300 s in open arms of the elevated plus-maze (**Figure 1A**) and 148±4 s in the closed arm. Mice socially isolated for 2 weeks, followed by 2 weeks of vehicle treatment (Iso2+Veh2) spent substantially less time in the open arms compared to the closed arms. Socially isolated mice treated with DHM (Iso2+D2) resulted in greater time spent in the open arm when compared with untreated socially isolated mice. Mice socially isolated for 4 weeks and administered DHM for 2 weeks (Iso4+D2) spent 101±6 s in the open arm and 169±10 s in the closed arm. In contrast, mice with 2 weeks of DZ administration after 4 weeks of isolation (Iso4+DZ2) spent less time in the open arm and more time in the closed arm in comparison to DHM treatment group. These results suggest that social isolation increases anxiety levels, and is impacted by the timeframe of social isolation. Furthermore, our data suggest that DHM administration ameliorates isolation-induced anxiety behavior, as observed with increased entry into and stay in the open arms. On the other hand, DZ treatment in the doses tested resulted in partial improvement in anxiety levels in these conditions but less effective than DHM.

To further examine anxiety-like behavior, we analyzed the distance traveled of the mice in the open field test (**Figure 1B**). During the 10 min observation, group-housed mice (G2+Veh2) traveled further distances, while mice housed in isolation (Iso2+Veh2) demonstrated a significant reduction in average path length suggesting that social isolation decreased motor activity of these mice (**Table 1**; **Figure 1B**). The additional 2-week treatment of DHM increased path lengths in the 2 and 4 weeks isolated mice (Iso2+D2 and Iso4+D2), attenuating the social isolation-induced reduction in locomotion (**Table 1**; **Figure 1B**). In contrast, DZ did not affect locomotor activity of socially isolated mice, as observed with their reduced distance traveled. The number of rearings and time spent in the center of the open field was significantly decreased in mice housed in isolation compared to that of group-housed mice (**Table 1**; **Figure 1B**). The duration of freezing behavior (initial time, when first placed into the apparatus), duration of tail lifting, and time mice spent in the corners were increased compared to group-housed mice (**Table 1**; **Figure 1B**). Administration of DHM in mouse groups Iso2+D2 and Iso4+D2 increased the number of rearings and the time in the center while decreasing the freezing, tail up and stay in the corner duration (**Table 1**; **Figure 1B**). In contrast, DZ did not improve the rearings, time in center, or freezing time of socially isolated mice (**Table 1**), though a decrease was noted for tail lift duration and time stay in the corners (**Table 1** and **Figure 1B**). Collectively, these results suggest that isolation decreases exploratory/locomotor activity in adult male C57BL/6J mice and that DHM treatment ameliorates these behavioral responses in socially isolated mice at both time points. For comparison, DZ treatment showed minimal effects in these social isolation-induced alterations in behavioral parameters.

**Table 1:** Analyses of various behavior details during open field test.

Group	Initiate time (s)	Tail up (s)	Rearing (# of times)	Path Length (cm)	Center (s)	Corner (s)
G2+Veh2	5.2 ± 0.2^†^	6.7 ± 1.1^†^	53.8 ± 1.5^†^	3835 ± 156.9^†^	44.2 ± 2.2^†^	26.2 ± 2.7^†^
G2+D2	4.7 ± 0.4^†^	5.8 ± 0.6^†^	56.7 ± 3.3^†^	3869 ± 136.7^†^	47.8 ± 6.6^†^	27.5 ± 6.6^†^
Iso2+Veh2	26.0 ± 1.3*	163.8 ± 6.1*	30.7 ± 2.8*	2710 ± 150.8*	3.0 ± 1.6*	116.7 ± 7.1*
Iso2+D2	8.7 ± 0.5*^†^	20.3 ± 3.2^†^	50.5 ± 6.8^†^	3355 ± 120.1*^†^	32.0 ± 5.1*^†^	26.8 ± 4.0^†^
Iso4+D4	9.2 ± 0.5*^†^	26.7 ± 4.4*^†^	45.0 ± 5.3^†^	3050 ± 123.8*	22.7 ± 2.5*^†^	38.3 ± 4.4^†^
Iso4+DZ2	27.8 ± 1.9*	60.0 ± 7.1*^†^	30.8 ± 2.5*	2660 ± 104.8*	10.0 ± 7.7*	35.2 ± 4.7^†^

Quantifications of anxiety-like behavior in socially isolated mice and DHM treatment. Initial time (freezing): the time duration from when the mouse first placed into the center of the apparatus to start moving. Tail up: the time of tail lifting. Rearing: total number of times of rearings. Pathlength: total distance of moving. Center: the total time duration the mouse stayed in the center 20x20 cm squares. Corner: the total duration the mouse stayed in the 4 corner 10x10 cm squares. *, p ≤ 0.05 vs. vehicle group housing control. †, p≤ 0.05 vs. Iso2+Veh2, (n = 6/group, one-way ANOVA followed by multiple comparison, Holm-Sidak method).

### DHM Reverses Isolation-Induced Impairment in GABAergic Neurotransmission.

**Figure 2:**
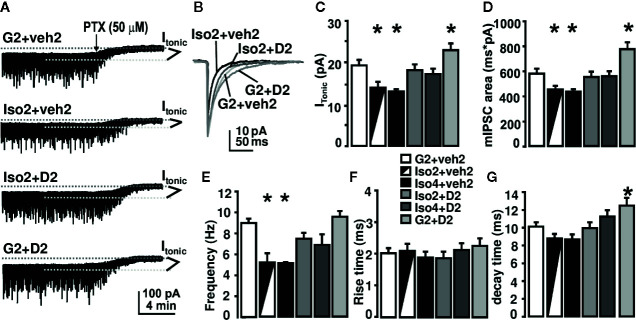
DHM reverses social isolation-induced impairment in GABA_A_R-mediated neurotransmission. **(A)** Sample recording traces from different groups; **(B)** superimposed mIPSC peaks from different treatment groups. Picro: application of 50 μM of picrotoxin. GABA_A_R-mediated I_tonic_ was calculated as the difference in holding current between the presence and absence of picrotoxin. **(C)** Summary of tonic currents from each group. **(D**–**G)** Summary of mIPSC kinetics. One-way ANOVA followed by multiple comparison, Sidak method. For tonic current, F(5, 64) = 8.62, p < 0.001. For mIPSC frequency, F(5, 63) = 9.0, P < 0.001. For rise time, F(5, 63) = 0.38, P = 0.86. For decay time, F(5, 64) = 5.76, P < 0.001. For mIPSC area, F(5, 63) = 9.62. P < 0.001. *, *p* ≤ 0.05 vs. group-housing control (G2 + Veh2); n = 4 mice/group; there could be multiple whole-cell patch recordings per mouse.

To gain insight into the underlying mechanisms of DHM effects on anxiety induced by social isolation, we measured GABA_A_R mediated whole-cell currents in DG granule cells of mouse hippocampal slices (**Figure 2**). We used picrotoxin (bath application, 50 mg/kg) to evaluate the magnitudes of I_tonic_ during these recordings. In mice group-housed (as control) for 2 weeks, followed by 2 weeks of vehicle treatment (G2+Veh2), I_tonic_ was 19±1.5 pA, and the frequency and area of mIPSCs were 8.8±0.39 Hz and 575.4±36.1 ms*pA, respectively. Social isolation for 2 weeks, followed by 2 weeks of vehicle treatment (Iso2+veh2)(total isolation was 4 weeks) decreased I_tonic_ to 13.6±1.28 pA, and reduced the frequency and area of mIPSCs to 5.0±0.98 Hz and 445.6±31.3 ms*pA, respectively. The decay time of mIPSC was not affected by isolation. Social isolation for 2-weeks, followed by 2-weeks of DHM treatment (Iso2+D2), significantly attenuated the effects observed in isolated mice such as I_tonic_, frequency, and area of mIPSCs (**Figure 2**). DHM administration alone (G2+D2) increased the decay time of mIPSCs to 12.5±0.5 ms (**Figure 2D**). The social isolation-induced reduction of I_tonic_, frequency, and area of mIPSCs suggest impairments of GABAergic transmission, including pre-synaptic GABA release, post- and extra-synaptic GABA_A_R function. DHM potentiates GABA_A_Rs and antagonizes the social isolation-induced impairments, which may be a mechanism underlying the therapeutic effects of DHM on isolation-induced anxiety-like behavior.

### DHM Reverses Social Isolation-Induced Reduction in Hippocampal ATP Levels and Gephyrin Protein Expression.

**Figure 3:**
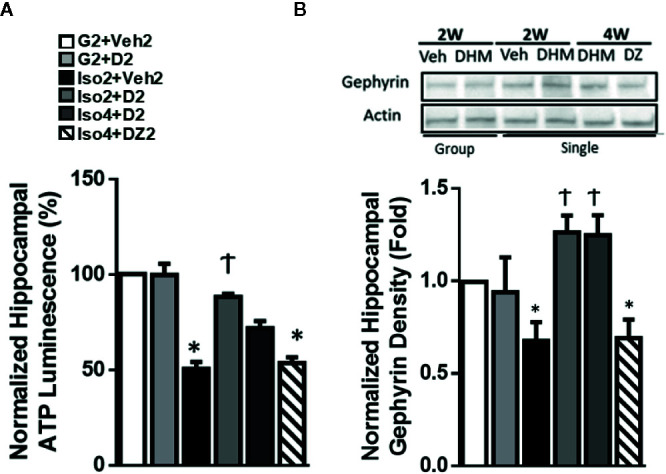
DHM restores hippocampal ATP levels and gephyrin protein expression induced by social isolation in mice. **(A)** Effects of social isolation and DHM treatment on ATP levels in microdissected hippocampi (nM/mg of tissue). One-way ANOVA followed by multiple comparison, Sidak method; F(5,18) = 9.999, p<0.0001. n=4/group. **(B)** Representative Western blot of gephyrin expression in hippocampal sections from different treatment groups. Group: group housing for 2w; Single: singly housed for 2w or 4w, followed by Veh (vehicle), DHM or DZ treatment for 2w. The Western blot image is representative of Western blots obtained from 3 different biological experiments and was cropped for saving space. Actin was used as loading control. One-way ANOVA followed by multiple comparison, Sidak method; F(5, 15) = 5.967, p<0.05. *p ≤ 0.05 vs. group housing control (G2 + Veh2). †*p* ≤ 0.05 vs. Iso2+Veh2.

Previous work reported decreases in ATP levels measured in the nucleus accumbens (NAc) of anxious rats (Hollis et al., 2015). This work linked lower ATP concentrations to anxiety, and this was attributed to a reduced mitochondrial function (Hollis et al., 2015). To investigate whether a similar mechanism could be identified in social isolation-induced anxiety mice, we next measured ATP levels from the hippocampal region that has been demonstrated to exhibit deficits under stressed conditions (Earnheart et al., 2007). Following social isolation (Iso2+Veh2), we found that ATP levels were reduced from 2.07 nM/mg to 1.397 nM/mg (approximately 32% of group-housed control mice) in the hippocampi (**Figure 3A**). Notably, administration of DHM in mice socially isolated restored the outcome of ATP levels to 1.871 (Iso2+D2) and 1.738 nM/mg (Iso4+D2) in the hippocampi, suggesting therapeutic effects of DHM on mitochondrial activity and ATP output (**Figure 3A**). However, DZ treatment (Iso4+DZ2 group) did not affect the social isolation-induced ATP reduction with our findings of 1.259 nM/mg (**Figure 3A**).

The scaffold protein, heat shock cognate 71 kDa protein (Hsc70), has been shown to interact with gephyrin, a scaffold protein essential for GABA_A_R clustering (Machado et al., 2011; Parra et al., 2016), via several mechanisms, including an ATP-dependent interaction. This ATP-dependent interaction is one of several mechanisms that may result in the modification of gephyrin expression and the associated GABA_A_R clustering. Therefore, to assess whether social isolation modified the expression of gephyrin and partially explains the changes in GABA_A_R neurotransmission, gephyrin protein expression was measured in extracted hippocampi and evaluated by Western blot. Gephyrin expression was 40% lower in isolated mice compared to group-housed mice (**Figure 3B**). DHM treatment restored gephyrin expression levels at both Iso2+D2 and Iso4+D2 groups relative to isolated mice without DHM. DZ treatment (Iso4+DZ2) had no effect on social isolation induced-reduction in gephyrin expression. Throughout the experimental period, gephyrin expression was positively correlated with ATP levels, suggesting that DHM pharmacologically restores ATP levels in socially isolated mice and, possibly ATP-dependently, restores gephyrin expression in the hippocampus, and subsequent GABA_A_R clustering and function (**Figure 2**).

## Discussion

The primary aim of the current study was to evaluate the anxiolytic properties of DHM and the cellular neurobiological mechanisms of the therapeutic effects of DHM using a mouse model in which anxiety is induced through social isolation. Isolation-induced changes in the CNS was examined using microdissected hippocampal tissue, a region of the brain showing compromised neurogenesis in anxiety (Earnheart et al., 2007). In this study, we found that social isolation of C57BL/6J mice (a) increased anxiety-like behavior; (b) impaired synaptic (phasic) and extrasynaptic (tonic) GABAergic inhibitory neurotransmission; (c) reduced hippocampal ATP levels and gephyrin expression, and (d) these behavioral deficits, and cellular and molecular impairments were attenuated with DHM administration. These results suggest that social isolation induces a reduction in intracellular ATP, gephyrin expression, and the plasticity changes in extra-, and postsynaptic GABA_A_Rs. In contrast, DHM antagonizes the molecular and cellular effects and improves the anxiety-like behavior induced by social isolation. Further investigations are necessary to determine whether these factors are critical to the development of chronic/repeated anxiety and whether these responses represent a novel treatment target. Therefore, through this study, we report that DHM is a potential candidate for prevention and pharmacotherapy for anxiety disorders. These results are in line with our previous findings of the anxiolytic effects of DHM in aged transgenic AD mice (TG2576 and TG-SwDI) (Liang et al., 2014a), while our electrophysiological and molecular results suggest a novel mechanism for DHM’s anxiolytic effects as outlined below.

Gephyrin is an extensively studied scaffolding protein at inhibitory synapses. Gephyrin was initially identified by its interaction with the glycine receptor, but more recent work has shown that it also interacts and recruits subunits of GABA_A_Rs (Wang et al., 1999; Combs and Markman, 2014). The function of gephyrin is regulated by protein phosphorylation and interactions with many proteins, such as neuroligin-2 and collybistin (D’Hulst et al., 2009). Additionally, the overall expression levels of gephyrin seem to indicate the total amount of GABA_A_Rs at a given time and have previously been utilized to evaluate the postsynaptic loss of GABAergic signaling, e.g., in the aging visual cortex (Yu et al., 2007; Pinto et al., 2010; Rozycka and Liguz-Lecznar, 2017). Although further research is necessary to clearly define the mechanisms, it is becoming apparent that the expression and function of gephyrin impact GABAergic neurotransmission and clustering. Therefore, we hypothesize that reductions in the expression of gephyrin may play a role in the reduced GABAaergic activity, plasticity of GABA_A_Rs, and neurobehavioral pathology and that these effects are ameliorated with DHM administration.

To test the hypothesis that DHM administration ameliorates anxiety and better understand the cellular mechanisms on gephyrin, we induced anxiety in mice via social isolation and measured anxiety-like behavior. Elevated-Plus maze and open field tests were conducted to assess anxiety-like responses, as these tests have been validated and are reliable for determining anxiety in rodent species of various strains without including noxious stimuli. Additionally, these behavioral methods are effective in evaluating anxiolytic drug effects with strain-specific responses between treatments and protocols (Pellow et al., 1985; Prut and Belzung, 2003; Barnett, 2007; Walf and Frye, 2007). Due to the nocturnal nature of mice, behavior testing was conducted during their active period (the dark phase of 12/12-h light/dark cycle) to represent a more accurate assessment and minimize interference of their circadian cycle. Circadian cycle affects motor activity in mice, with less activity and movement in the light phase (Ma et al., 2011; Koskela et al., 2020). Additionally, mice are considered insensitive to red light (Peirson et al., 2018). The intensity and color of light are additional variables that contribute to changes in behavior and stress (McReynolds et al., 1967; Kapogiannatou et al., 2016). Therefore, the use of dim red light in our studies is expected to minimally affect mouse behavior compared to bright light stimuli.

Animals were treated either with DHM, vehicle, or DZ, and anxiety-like behavior was evaluated using an elevated plus maze and open field test. We found that the single-housed mice displayed anxiety-like behavior and reduced locomotion (**Figure 1**). Our findings of reduced locomotion in socially isolated mice differ from previous reports that have observed no changes in locomotion of isolated male C57BL/6J mice (Varty et al., 2006; Lander et al., 2017). Factors, such as object deprivation and reduced visibility with opaque cages during isolation, the time of testing (dark phase vs. light phase) during circadian cycle, and lighting conditions during testing may have induced variable behavioral outcomes in mice.

Isolated mice that were provided DHM showed a significant improvement in anxiety-like behavior and locomotion that is similar to group-housed mice. When DZ was administered to socially isolated mice (Iso4+DZ2), we found minimal anxiolytic effects. This is likely due to the plasticity of GABA_A_Rs after social isolation (Rodgers and Shepherd, 1993; Pinna et al., 2006; Rudolph and Knoflach, 2011; Lindemeyer et al., 2017) and might be related to the impact of social deprivation on the psychoemotional status and underlying physiological factors. Previous investigations have reported variable DZ responses, such as reduced efficacy and GABA_A_R modulation, relating to physiological and psychoemotional factors (Wongwitdecha and Marsden, 1996; Ojima et al., 1997; Serra et al., 2000; Kudryavtseva and Bondar’, 2002; Pinna et al., 2004; Matsumoto et al., 2005). The administration of DZ at 1.42 and 1.98 mg/kg in socially isolated mice increased locomotor activity in adult male Swiss-Webster mice, potentially due to the stimulation of the induced overexpression α5-containing GABA_A_R subtypes (Pinna et al., 2006). Our results showed minimal effect of DZ (10 mg/kg) on locomotor activity of socially isolated C57BL/6J mice relative to group-housed mice or DHM treatment, in which social isolation has previously been reported to modify these outcomes in studies using anxious C57BL/6J mice. This lack of effect may be due to the DZ dose on GABA_A_R function and activity on differentially expressed GABA_A_R subtypes induced by social isolation. The GABAergic plasticity that influenced DZ pharmacological activity on anxiety and locomotion in this study did not affect the pharmacological activity of DHM, in which DHM positively modulates GABA_A_Rs (Shen et al., 2012), and we anticipated that these effects were beneficial to its anxiolytic properties. Our findings of the therapeutic effects of DHM suggest alternative and novel medication effects in this mouse model when compared against DZ. These effects are consistent in reducing anxiety-like behavior regardless of the physiological and psychoemotional state that negates DZ-mediated responses in socially isolated mice, suggesting a novel anxiolytic mechanism. The pharmacological activity of DHM in socially isolated C57BL/6J mice should be further assessed to determine the GABAergic plasticity that altered DZ activity and expand upon the alternative mechanisms of anxiolytic properties related to ATP and gephyrin recovery. The limitations of certain control groups (time-matched controls and added treatment controls) in this study are critical to elucidate these changes in DZ activity, and better illustrate the novel mechanisms of DHM. To thoroughly investigate the observed DZ treatment resistance, ongoing studies are utilizing DZ, and other anxiolytics to provide insight into additional mechanisms of DHM with added controls for both 4-week and 6-week time points.

Extrasynaptic GABA_A_Rs are composed of receptor subunits that convey biophysical properties ideally suited to the generation of persistent inhibition and are pharmacologically and functionally distinct from their synaptic counterparts (Liang et al., 2009). Our previous studies have demonstrated that GABA_A_R-mediated I_tonic_ plays a major role in the physiological response to sedative/anesthetic/neurosteroid drugs (Liang et al., 2004a; Liang et al., 2007). These plastic responses of I_tonic_ can be attributed to changes in extrasynaptic GABA_A_R subunit and functions (Liang et al., 2009). Although other investigations have reported that social isolation induced an increase in the GABAergic tonic current in male Sprague-Dawley rats (Serra et al., 2006), our study reports a reduction of I_tonic_ and charge transfer (area) of mIPSCs in the DG granule cells of male C57BL/6J mice. The discrepancy in I_tonic_ is likely due to the different rodent models, i.e., 30-d-old rats vs. adult mice and/or experimental settings, i.e., the recording temperature was room temperature in Serra et al. (2006) vs. 35°C used in this study. With the administration of DHM, we observed a reversed outcome in male C57BL/6J mice, resulting in higher measures in frequency and charge transfer of mIPSCs and I_tonic_ potentially contributing to the reduction in anxiety-like behavior (**Figure 1**). Future investigations are necessary to confirm the correlations between these responses and the GABAergic circuit plasticity.

With recent findings of suboptimal mitochondrial function in the NAc in high-anxious rats (Hollis et al., 2015), we were motivated to further investigate the cellular and molecular mechanisms of DHM’s anxiolytic effects on mitochondrial ATP production in social isolation mice that display anxiety-like behavior. We were interested in determining whether similar outcomes of suboptimal ATP production occurred in our isolated mice that exhibit anxiety and whether DHM is able to modify these outcomes. In our study, we found reduced ATP levels in the microdissected hippocampi of isolated mice (**Figure 3**). Mice that were socially isolated and orally administered DHM had higher hippocampal ATP levels than their untreated counterparts, with levels comparable to that of group-housed mice. Our findings illustrate the beneficial molecular effects of DHM on mitochondrial ATP output in the hippocampus of socially isolated mice that exhibit anxiety, suggesting a potential molecular mechanism of DHM related to its anxiolytic activity. These findings are consistent with other investigations that have found that DHM administration increases the ATP content of HT-22 mouse hippocampal neuronal cells via increased activity of complex I, II, and ATP synthase (Liu et al., 2016). These findings from Liu and colleagues reported that these DHM-mediated effects were associated with sirtuin-3 activity and downstream mitochondrial biogenesis and improved mitochondrial morphology in response to hypoxia in male Sprague Dawley rats (Liu et al., 2016). These mechanisms have also been reported in other tissues, such as skeletal muscle of male 129/SvJ mice (Shi et al., 2015). Furthermore, DHM has been found to activate the expression and activity of sirtuin-1 and downstream activation of Pparg coactivator 1 alpha (PGC-1α), resulting in enhanced mitochondrial function and biogenesis in the livers of 129S1/SvImJ mice (Zeng et al., 2019). Future studies are necessary to determine whether this DHM-mediated ATP conservation is related to similar outcomes in mitochondrial biogenesis, activity, and ATP production.

A mechanism of scaffold interaction of Hsc70 with gephyrin and subsequent GABA_A_R clustering is suggested to be dependent on ATP concentrations (Combs and Markman, 2014; Karim et al., 2018). Due to the changes in ATP concentrations identified in the hippocampi of socially isolated mice, we next wanted to investigate whether an associated reduction of gephyrin expression was observed with social isolation. We found that gephyrin protein expression was decreased in the hippocampi of socially isolated mice in parallel to the reduced ATP levels after 2- and 4-week social isolation, followed by an additional 2 weeks of treatment and isolation (**Figure 3A**). In contrast, mice with DHM treatment, while demonstrating higher levels of ATP and a significant reduction in anxiety-like behavior, showed a higher expression of gephyrin protein in the hippocampus, suggesting that DHM restores ATP levels and maintains the expression of gephyrin in isolated mice (**Figure 3B**). Furthermore, these sustained levels of gephyrin may contribute to the restored GABAergic neurotransmission in the DG granule cells (**Figure 2**). Evidence of neuroplastic changes on gephyrin would suggest that DHM provided not only acute symptom relief but also long-lasting plastic changes of anxiety circuits, a substantial paradigm shift for the treatment of anxiety. Therefore, our investigations have identified that DHM has positive GABA_A_R modulatory effects that partly explains its anxiolytic properties, meanwhile, illustrating additional indirect or direct responses that restore gephyrin protein expression and ATP levels. The combination of these effects, along with other potential downstream responses, can provide long-term anxiolytic benefits in socially isolated mice that were resistant to DZ treatment. Current studies are underway to determine the benefits of DHM administration in remodeling anxiety circuits and its potential in alleviating downstream related cognitive decline.

Of note, unlike BZs, such as DZ, which induce a loss-of-righting-reflex at 20 mg/kg (Liu et al., 2016), DHM induces loss-of-righting-reflex only when dosed hundreds-fold higher than its pharmacological dose for depression. This suggests that DHM will show a more benign side effect profile within its intended dosage for anxiolytic properties, supporting its safety profile for the use as an anxiolytic. Hence, DHM may have a broader application in the long-term pharmacotherapy of anxiety and other chronic disorders, while reducing the potential for adverse side effects.

## Data Availability Statement

The raw data supporting the conclusions of this article will be made available by the authors, without undue reservation.

## Ethics Statement

The animal study was reviewed and approved by University of California (UCLA) Institutional Animal Care and Use Committee.

## Author Contributions

JS, AS, YS, XS, RO, DH, and JL designed research. JS, AS, YS, XS, and JL performed the experiments. JS, AS, YS, XS, and JL analyzed data. JS, AS, XS, RO, DD, DH, and JL wrote the paper.

## Funding

This work was supported by the National Institute of Health grants AA017991 (to JL), AA07680 and AA021213 (to RO), AA022448 (to DD), R01HL135623-01, R42DA044788-02 (to XS), and American Foundation for Pharmaceutical Education (AFPE; to JS) and Carefree Biotechnology Foundation.

## Conflict of Interest

The authors declare that the research was conducted in the absence of any commercial or financial relationships that could be construed as a potential conflict of interest.

## Abbreviations

AD, Alzheimer’s disease; BZs, benzodiazepines; DZ, diazepam; DHM, dihydromyricetin; GABA_A_Rs, GABA_A_ receptors; I_tonic_, tonic current; mIPSCs, miniature inhibitory postsynaptic currents.
