# Supplementary figures and images for: Modulation of Hippocampal GABAergic Neurotransmission and Gephyrin Levels by Dihydromyricetin Improves Anxiety
Source: Front Pharmacol. 2020 Jul 9;11:1008. doi: 10.3389/fphar.2020.01008 (PMC7364153; doi:10.3389/fphar.2020.01008)

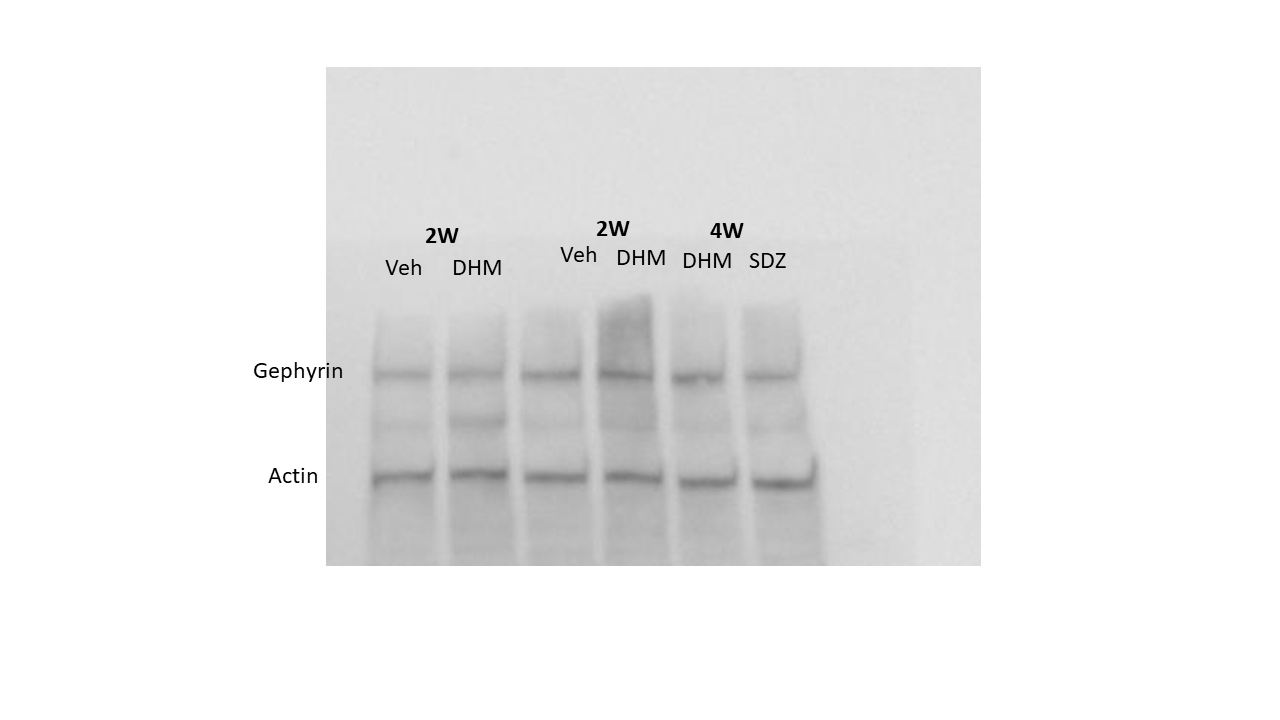

Supplement: Supplementary file 1 [file Image_1.tif]
